# Transcriptional Regulation of HSP60, HSP70, and HSP90 in Response to Selenium Nanoparticles and Sodium Selenite in Periparturient Cloned Saanen Goats and Their Offspring

**DOI:** 10.1002/vms3.71135

**Published:** 2026-07-29

**Authors:** Gholam‐Ali Kojouri, Hossein Hassanpour, Ahmad Yousefi

**Affiliations:** ^1^ Department of Clinical Sciences Faculty of Veterinary Medicine Shahrekord University Shahrekord Iran; ^2^ Department of Basic Science Faculty of Veterinary Medicine Shahrekord University Shahrekord Iran; ^3^ Health Equity Research Center Shahed University Tehran Iran; ^4^ Graduate of Large Animal Internal Medicine Faculty of Veterinary Medicine University of Shahrekord Shahrekord Iran

**Keywords:** cloned goat, heat shock protein, Selenium nanoparticle, selenium

## Abstract

**Objectives:**

This study investigated the effects of selenium supplementation in the form of selenium nanoparticles (SeNPs) and sodium selenite (SSe) on the transcriptional regulation of heat shock proteins (HSP60, HSP70, and HSP90) in periparturient cloned and non‐cloned Saanen goats and their offspring.

**Methods:**

A total of 24 pregnant goats (12 cloned via somatic cell nuclear transfer and 12 non‐cloned) were divided into three groups: SeNPs, SSe and control. Supplementation was administered orally from 21 days before expected parturition. Serum selenium levels and HSP gene expression were measured at multiple time points relative to parturition.

**Results:**

Both SeNPs and SSe significantly increased serum selenium levels, with SeNPs demonstrating superior bioavailability. Supplemented groups exhibited significantly lower transcriptional levels of HSP60, HSP70 and HSP90 during the periparturient period, with SeNPs showing a more potent suppressive effect. A significant negative correlation was observed between serum selenium and HSP transcription. No differences were found between cloned and non‐cloned animals in selenium levels or HSP expression.

**Conclusions:**

These findings suggest that selenium supplementation, particularly as SeNPs, enhances antioxidant capacity and reduces cellular stress, as reflected by the downregulation of HSP genes. This study highlights the potential of nano‐selenium to improve stress resilience during the critical transition period in goats and their offspring, irrespective of cloning status.

## Graphical abstract

Selenium nanoparticles (SeNPs) outperform sodium selenite in raising serum selenium levels and suppressing HSP60, HSP70, and HSP90 transcription in periparturient goats and their offspring, indicating reduced cellular stress regardless of cloning status.

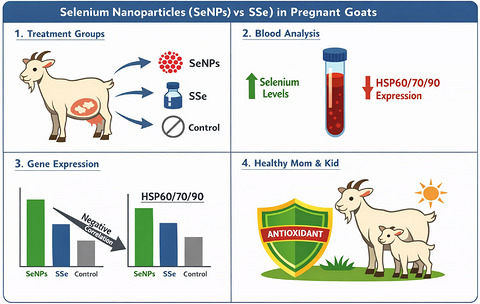

## 1 Introduction

The transition period, spanning the final weeks of gestation to the early weeks of lactation, represents a physiological and metabolic challenge for ruminants, characterised by significant endocrine changes and heightened susceptibility to oxidative stress (Kour et al. 2022). In cloned animals produced via somatic cell nuclear transfer (SCNT), these challenges are often exacerbated. While SCNT is a powerful biotechnology, it is associated with a high incidence of prenatal and perinatal losses, which are frequently attributed to placental dysfunction and aberrant foetal development. A critical factor underlying these complications is the inadequate cellular stress response in SCNT‐derived conceptuses, which may be linked to errors in epigenetic reprogramming (Manna et al. 2022).

Heat shock proteins (HSPs), particularly the major families HSP60, HSP70 and HSP90, are highly conserved molecular chaperones that play a fundamental role in cellular proteostasis (Hu et al. 2022). They facilitate the proper folding of nascent polypeptides, prevent the aggregation of denatured proteins, and are rapidly upregulated in response to a variety of stressors, including heat, oxidative insults and inflammatory signals (Singh et al. 2024). The expression profile of these HSPs serves as a sensitive biomarker for cellular stress. In the context of pregnancy, especially in the demanding transition period and in the physiologically distinct state of cloned pregnancies, the regulation of HSPs is crucial for maintaining cellular integrity and ensuring normal foetal development (Swelum et al. 2020).

Selenium is an essential trace element with a well‐established role as a potent antioxidant, primarily through its incorporation into a family of selenoproteins, most notably glutathione peroxidase and thioredoxin reductase (Wang et al. 2025). These enzymes are critical components of the body's endogenous defence system, neutralising reactive oxygen species (ROS) and mitigating oxidative damage to cellular structures. The physiological demand for selenium becomes profoundly magnified during periods of high metabolic activity, such as late gestation and parturition (Hubeni et al. 2024). In the pregnant dam, this heightened demand is driven by the needs of the developing foetus, the expansion of maternal tissues and the immense oxidative burst associated with the process of labour itself (Grzeszczak et al. 2023). Adequate selenium status is crucial not only for protecting maternal tissues but also for ensuring proper foetal development, particularly of the neuromuscular and immune systems. Furthermore, selenium is vital for the neonate, who is born with limited antioxidant capacity and is highly susceptible to oxidative stress (Kojouri et al. 2020, Sattar 2021). The transfer of selenium across the placenta is the primary mechanism for building foetal reserves, which are essential for supporting the neonate through the critical first days of life before the intake of selenium‐rich colostrum. However, the efficacy of selenium supplementation depends heavily on its form (Hogan and Perkins 2022). Traditional inorganic sources, such as sodium selenite (SSe), have limitations, including lower bioavailability due to complex interactions in the rumen and potential pro‐oxidant effects at higher doses. Recent advancements in nutritional science have introduced selenium nanoparticles (SeNPs) as a superior alternative (Sadeghian Chaleshtori et al. 2017). SeNPs exhibit higher bioavailability, enhanced absorption and significantly lower toxicity. Their nanoscale size and unique physicochemical properties allow for more efficient passage through biological membranes, leading to a more effective and sustained increase in serum and tissue selenium levels (Surai and Cardoso 2024).

It is hypothesised that this nutritional intervention may modulate the cellular stress response. This is supported by findings that selenium nanoparticle supplementation improved haematological parameters in periparturient Saanen goats and their offspring compared to sodium selenite, highlighting the superior bioavailability and physiological efficacy of the nanoform (Yousefi et al. 2021). However, the impact of selenium supplementation, particularly in its nanoform, on the transcriptional regulation of key stress markers like HSPs in pregnant cloned goats and their offspring remains unexplored. Therefore, this study aims to monitor the transcription rates of HSP60, HSP70 and HSP90 genes in pregnant cloned Saanen goats and their offspring in response to oral supplementation with SeNPs and SSe during the critical transition period. Elucidating this relationship will provide deeper insights into the molecular mechanisms by which nano‐selenium can enhance stress resilience and potentially improve the survival and health outcomes of SCNT‐cloned animals and their progeny.

## 2 Materials and Methods

### 2.1 Animals and Experimental Design

A total of 24 pregnant Saanen goats were selected for this study, comprising 12 cloned (produced via Somatic Cell Nuclear Transfer using abattoir‐derived oocytes) and 12 non‐cloned animals. All goats were housed under identical conditions at the Royan Institute for Biotechnology, Isfahan. Following health assessment and ultrasonographic confirmation of pregnancy, the goats were randomly allocated into three experimental groups (*n* = 8 per group; each containing 4 cloned and 4 non‐cloned animals): (1) SeNPs group, received oral supplementation of selenium nanoparticles at 0.05 mg/kg (pure selenium dose, 0.05 mg/kg) body weight; (2) SSe group, received oral supplementation of sodium selenite at 0.05 mg/kg body weight (pure selenium dose, 0.023 mg/kg); and (3) control group, received an equivalent volume of distilled water. The supplementation was administered daily for 10 days, commencing 21 days before the expected parturition date. The dose of 0.05 mg/kg body weight was selected based on a previous study in Saanen goats (Yousefi et al. 2021), which demonstrated that this level effectively elevated serum selenium without signs of toxicity. The 10‐day duration and initiation 21 days prepartum were chosen to cover the critical periparturient period when oxidative stress peaks, while avoiding prolonged supplementation that might lead to selenium accumulation above physiological ranges.

### 2.2 Preparation of Selenium Nanoparticles

Selenium nanoparticles (SeNPs) were synthesised according to the redox reaction method described previously (Zhang et al. 2023). In brief, an aqueous solution of selenium dioxide (SeO_2_) was reduced using ascorbic acid. The formation of red‐coloured SeNPs, indicating the reduction of Se^4^^+^ ions to elemental selenium, was confirmed. The synthesized nanoparticles were characterised to be amorphous and monoclinic, with a size range of 80–150 nm. Characterisation was performed using dynamic light scattering and transmission electron microscopy to confirm size and morphology (data not shown).

### 2.3 Blood Sampling, RNA Extraction and cDNA Synthesis

Blood samples were collected from 24 goats (8 dams per group) via the jugular vein at multiple time points: Days −14, −7, 0 (parturition), +7 and +14 relative to kidding. For the 24 offspring (8 neonates per group), blood samples were taken at birth (Day 0), Day 7 and Day 14 of life.

Total RNA was extracted from the leukocyte‐rich buffy coat layer of blood samples using the RNX‐PLUS solution (SinaClon, Iran), following the manufacturer's instructions. All RNA samples had A260/A280 ratios between 1.9 and 2.1, and integrity was confirmed by agarose gel electrophoresis showing distinct 18S and 28S ribosomal bands. To ensure high‐purity RNA, any potential genomic DNA contamination was removed using a DNase treatment step.

Complementary DNA (cDNA) was synthesized from the extracted RNA using the Prime‐Script RT Reagent Kit (Takara Bio, Japan). The reaction mixture included RNA template, reverse transcriptase enzyme, Oligo dT primers and random hexamers. Each cDNA synthesis reaction included a no‐reverse‐transcriptase control to rule out genomic DNA contamination. The synthesized cDNA was stored at −20°C for subsequent gene expression analysis.

### 2.4 Quantitative Real‐Time PCR (qRT‐PCR)

The transcriptional levels of *HSP60*, *HSP70* and *HSP90* were quantified using quantitative real‐time PCR. *GAPDH* was used as the reference gene for normalization. Specific primers for the target and reference genes were designed using NCBI's primer‐blast tool. To confirm species specificity, each primer pair was aligned against the caprine (*Capra hircus*) genome using BLAST. No significant homology with other ruminant or bacterial sequences was found. Melting curve analysis for each amplicon produced a single peak, and PCR products were run on 2% agarose gels to verify the expected band sizes (103 bp for *HSP60*, 77 bp for *HSP70*, 85 bp for *HSP90*, 109 bp for *GAPDH*). The primer sequences and their accession numbers are listed in Table 1 of the original document. The reactions were performed in a Rotor‐Gene Q 6000 thermocycler (Qiagen, USA) using SYBR Green Premix (Takara Bio). The thermal cycling protocol consisted of an initial denaturation at 95°C for 30 seconds, followed by 40 cycles of denaturation at 94°C for 40 s, annealing at 58°C–60°C for 20 s, and extension at 72°C for 20 s. Relative gene expression was calculated using the Pfaffl method, which accounts for PCR amplification efficiency. Each qRT‐PCR reaction was performed in triplicate technical replicates for each biological sample. The mean Ct value of the three technical replicates was used for subsequent calculations. No‐template controls were included in each run to detect contamination. Inter‐run variability was monitored using a calibrator sample run on every plate; the coefficient of variation for Ct values of the calibrator across all plates was < 2%. The ratio of the expression of each target gene (*HSP60*, *HSP70* and *HSP90*) relative to the reference gene (*GAPDH*) was determined for both treatment and control groups.

### 2.5 Measurement of Serum Selenium Concentration

The concentration of selenium in blood serum was quantified to monitor the bioavailability of the supplemented selenium forms. Serum was separated from clotted blood samples by centrifugation. Selenium levels were measured using a Perkin Elmer 4100 atomic absorption spectrometer. Briefly, 2 mL of serum was mixed with 1 mL of a 5% ammonium pyrrolidine dithiocarbamate solution and shaken for 20 minutes to form organometallic complexes. Subsequently, 1 mL of methyl isobutyl ketone was added, and after 10 minutes, the mixture was centrifuged at 2500 rpm to separate the organic phase. Following instrument calibration with standard solutions, the selenium content in the samples was determined using the Palladium Matrix Modifier and Win Lab 32 software (Ambarak and Asweisi 2020).

### 2.6 Statistical Analysis

Statistical analysis was performed using SPSS software (Version 16). Data are presented as mean ± standard error of the mean (SEM). To evaluate the effects of treatment, time and cloning status, a repeated measures ANOVA was used, followed by a Tukey post‐hoc test for multiple comparisons. Prior to repeated measures ANOVA, the assumption of sphericity was assessed using Mauchly's test. When sphericity was violated (*p < *0.05), the Greenhouse–Geisser correction was applied to adjust the degrees of freedom. Normality of residuals was assessed using the Shapiro–Wilk test, and homogeneity of variances was verified using Levene's test. Outliers were identified by boxplot inspection and studentised residuals (threshold ± 3). No significant outliers were removed. A Pearson correlation analysis was conducted to assess the relationship between serum selenium levels and gene expression. A *p*‐value of less than 0.05 was considered statistically significant.

## 3 Results

### 3.1 Serum Selenium Levels

In the SeNP and SSe groups, serum selenium levels showed a significant increase from Day −14 to peak at Days −7 and 0 (parturition), maintaining elevated levels through Day +7 (*p* < 0.05). The SeNP group consistently demonstrated the highest concentrations across these time points, followed by the SSe group, with both treatment groups showing significantly higher levels than the control group (*p < *0.05). By Day +14, serum selenium levels in all treatment groups had returned to baseline values comparable to the control group (Table 2).

**TABLE 1:** Primers used in cloned and non‐cloned goats.

Target	Primers	Accession No.	Melting temperature	PCR product
HSP60	5′‐CTCCTCATCTCACTCGGGCT‐3′	XM_004004792.3	60.75	103 bp
	5′‐ACGGCTACAGCATCGGCT‐3′		61.46	
HSP70	5′‐GCGTGATCAACGACGGAGAC‐3′	JN604434.1	61.40	77 bp
	5′‐CTCCTCTGGGTAGAACGCCT‐3′		60.68	
HSP90	5′‐CTCCTCTGGGTAGAACGCCT‐3′	XM_004017995.3	62.40	85 bp
	5′‐CCTGGAAGGAGACGACGACAC‐3′		61.64	
GAPDH	5′‐CAGAGAACGGGAAGCTCGTC‐3′	NM_001190390.1	60.46	109 bp
	5′‐GACTCCACCACGTACTCAGC‐3′		60.11	

**TABLE 2:** Selenium levels across different groups and days.

Periods	Control	SeNPs	SSe
**Dams (µg/dL)**			
Day −14	19.0 ± 0.37	21.2 ± 0.17^a^	21.1 ± 0.19^a^
Day −7	19.2 ± 0.70^B^	44.3 ± 0.20^Ab^	44.1 ± 0.18^Ab^
Day 0 (Parturition)	17.8 ± 0.25^C^	43.1 ± 0.26^Ab^	37.6 ± 0.05^Bc^
Day +7	18.8 ± 0.69^C^	41.5 ± 0.24^Ab^	34.3 ± 0.05^Bc^
Day +14	18.8 ± 0.40^B^	19.7 ± 0.36^Aa^	18.5 ± 0.08^Ba^
**Neonates (µg/dL)**			
Day 0 (Birth)	16.8 ± 0.11^Ca^	30.4 ± 0.08^Aa^	26.6 ± 0.09^Ba^
Day +7	18.9 ± 0.17^Cb^	30.5 ± 0.06^Aa^	24.4 ± 0.04^Bb^
Day +14	19.1 ± 0.16^Ab^	19.1 ± 0.03^Ab^	18.5 ± 0.08^Bc^

*Note*: Data are as mean ± standard error.

^A, B, C^Significant difference within a row across experimental groups (*p < *0.05).

^a, b, c^Significant difference within a column across days within a group (*p < *0.05).

A similar temporal pattern was observed in offspring throughout the neonatal period. At birth (Day 0), kids from the SeNP group exhibited the highest serum selenium levels, followed by the NaSe group, with both treatment groups showing significantly higher concentrations than the control group (*p < *0.05). This pattern persisted through Day +7, though selenium levels began to decline in all groups. By Day +14, serum selenium concentrations had equalised across all groups, with no significant differences observed between treatment and control offspring (Table 2).

### 3.2 Transcriptional Regulation of HSP Genes in Goats

In the control group, a marked upregulation of all three HSP genes was initiated by Day −7, culminating in a significant peak on the day of parturition (Day 0). For HSP60 and HSP90, this state of elevated transcription persisted through Day +7, before returning to baseline levels by Day +14 (Table 3).

**TABLE 3:** HSPs gene transcription (/GAPDH) in leukocytes of goats across different groups and days.

Periods	Control	SeNPs	SSe
**HSP60**			
Day −14	1.66 ± 0.009^a^	1.66 ± 0.006^a^	1.66 ± 0.008^a^
Day −7	1.67 ± 0.004^a^	1.66 ± 0.004^a^	1.66 ± 0.005^a^
Day 0 (Parturition)	1.85 ± 0.010^Bb^	1.73 ± 0.010^Ab^	1.73 ± 0.005^Ab^
Day +7	1.85 ± 0.005^Bb^	1.73 ± 0.004^Ab^	1.74 ± 0.007^Ab^
Day +14	1.67 ± 0.007^a^	1.67 ± 0.005^a^	1.67 ± 0.006^a^
**HSP70**			
Day −14	0.23 ± 0.005^a^	0.23 ± 0.005	0.23 ± 0.004^a^
Day −7	0.24 ± 0.006^a^	0.23 ± 0.005	0.23 ± 0.008^a^
Day 0 (Parturition)	0.38 ± 0.011^Cb^	0.24 ± 0.006^A^	0.25 ± 0.003^Bb^
Day +7	0.24 ± 0.002^a^	0.23 ± 0.006	0.24 ± 0.002^a^
Day +14	0.24 ± 0.004^a^	0.22 ± 0.007	0.22 ± 0.004^a^
**HSP90**			
Day −14	2.21 ± 0.012^a^	2.21 ± 0.008	2.21 ± 0.007a
Day −7	2.22 ± 0.009^a^	2.21 ± 0.007	2.22 ± 0.009a
Day 0 (Parturition)	4.28 ± 0.026^Cb^	2.22 ± 0.006^A^	2.53 ± 0.005^Bb^
Day +7	2.33 ± 0.007^Bc^	2.22 ± 0.003^A^	2.33 ± 0.012^Bc^
Day +14	2.22 ± 0.014^a^	2.21 ± 0.004	2.22 ± 0.003^a^

*Note*: Data are as mean ± standard error.

^A, B, C^Significant difference within a row across experimental groups for each HSP (*p < *0.05).

^a, b, c^Significant difference within a column across days within a group (*p < *0.05).

Throughout the periparturient period (Days −7, 0 and +7), the transcriptional levels of all three HSP genes in both the SeNP and SSe groups were significantly lower than those in the control group (*p < *0.05). The suppressive effect was most pronounced on the day of parturition. Furthermore, the SeNP treatment demonstrated a consistently superior efficacy compared to SSe, resulting in significantly lower transcription of HSP70 and HSP90 at critical time points, particularly on Day 0 and Day +7. By Day +14, the transcriptional levels of all HSP genes had normalised across all experimental groups, with no significant differences observed (Table 3).

### 3.3 Transcriptional Regulation of HSP Genes in Offspring

In newborns from the control group, the highest transcriptional levels for all three HSP genes were observed immediately at birth (Day 0). This was followed by a significant decline by Day +7, with levels continuing to decrease gradually until they reached their lowest point by Day +14 (Table 4).

**TABLE 4:** HSPs gene transcription (/GAPDH) in leukocytes of goat neonates across different groups and days.

Periods	Control	SeNPs	SSe
**HSP60**			
Day 0 (Birth)	1.83 ± 0.007^Ba^	1.66 ± 0.005^Aa^	1.67 ± 0.005^Aa^
Day +7	1.82 ± 0.015^Ba^	1.65 ± 0.004^Aa^	1.66 ± 0.004^Aa^
Day +14	1.51 ± 0.012^b^	1.50 ± 0.010^b^	1.50 ± 0.007^b^
**HSP70**			
Day 0 (Birth)	0.33 ± 0.005^Ca^	0.24 ± 0.001^Aa^	0.28 ± 0.002^Ba^
Day +7	0.27 ± 0.002^Bb^	0.22 ± 0.001^Ab^	0.26 ± 0.003^Bb^
Day +14	0.19 ± 0.003^c^	0.19 ± 0.003^c^	0.19 ± 0.001^c^
**HSP90**			
Day 0 (Birth)	3.81 ± 0.024^Ca^	2.13 ± 0.001^A^	2.57 ± 0.019^Ba^
Day +7	2.17 ± 0.007^b^	2.13 ± 0.002	2.16 ± 0.001^b^
Day +14	2.14 ± 0.001^b^	2.11 ± 0.001	2.12 ± 0.002^b^

*Note*: Data are as mean ± standard error.

^A, B, C^Significant difference within a row across experimental groups for each HSP (*p < *0.05).

^a, b, c^Significant difference within a column across days within a group (*p < *0.05).

At birth (Day 0) and throughout the first week of life (Day +7), kids born to selenium‐supplemented dams exhibited significantly lower transcriptional levels of all HSP genes compared to offspring from the control group (*p < *0.05). This effect was most pronounced in the group whose mothers received SeNPs, which consistently resulted in the lowest HSP transcript levels. By Day +14, the differences in gene expression between the treatment and control groups were no longer statistically significant, as transcript levels converged across all offspring (Table 4).

### 3.4 Correlation Between Serum Selenium and HSP Transcription

A significant negative correlation was found between serum selenium levels and the transcription of *HSP60*, *HSP70*, and *HSP90* in goats on the day of parturition. This inverse relationship was also present for *HSP60* and *HSP90* on Day +7 (Table 5).

**TABLE 5:** Pearson correlation between serum selenium levels and transcription of HSPs in leukocytes of goats and their neonates on different days.

Variables	Day −14	Day −7	Day 0(Parturition)	Day +7	Day +14
**Dams**					
Selenium/ HSP60	—	—	*r = *‐0.901 *p < *0.001	*r = *‐0.933 *p* = 0.027	—
Selenium/ HSP70	—	—	*r = *‐0.950 *p < *0.001	—	—
Selenium/ HSP90	—	—	*r = *‐0.996 *p < *0.001	*r = *‐0.762 *p < *0.001	—
**Neonates**					
Selenium/ HSP60	—	—	*r = *‐0.952 *p < *0.001	*r = *‐0.820 *p < *0.001	—
Selenium/ HSP70	—	—	*r = *‐0.942 *p < *0.001	*r = *‐0.859 *p < *0.001	—
Selenium/ HSP90	—	—	*r = *‐0.995 *p < *0.001	*r = *‐0.782 *p < *0.001	—

*Note: p < *0.05 means statistically significant.

In newborns, a significant negative correlation was observed between serum selenium levels and the transcription of all three HSP genes at birth and on Day +7 (Table 5).

### 3.5 Effect of Cloning Status

No significant differences were found in any of the serum selenium levels or the transcriptional profiles of *HSP60*, *HSP70* and *HSP90* between the cloned and non‐cloned goats or their offspring at any time point (Table S1).

## 4 Discussion

The findings of this study demonstrate that selenium supplementation, particularly in the form of selenium nanoparticles, effectively modulates the transcriptional activity of key heat shock proteins (*HSP60*, *HSP70* and *HSP90*) during the physiologically demanding periparturient period in Saanen goats and their offspring. The observed suppression of HSP transcription in supplemented animals, in conjunction with elevated serum selenium levels, is consistent with a role for selenium in enhancing cellular stress resilience, thereby reducing the need for a robust HSP‐mediated chaperone response.

Notably, the SeNPs and SSe groups received equal compound mass (0.05 mg/kg), resulting in different elemental selenium doses. Thus, the observed superiority of SeNPs reflects both higher selenium intake and potential nano‐specific advantages. While these factors cannot be fully disentangled from the present data, peak serum selenium levels in SeNPs were disproportionately higher than the input difference, suggesting that nano‐specific bioavailability advantages exist beyond the higher elemental dose.

The significant increase in serum selenium levels in both SeNPs and SSe groups, with SeNPs yielding consistently higher concentrations, aligns with a growing body of evidence supporting the superior bioavailability of nanoparticulate selenium (Pouri et al. 2018, Sadeghian et al. 2012). Previous studies have shown that SeNPs are absorbed more efficiently and exhibit a more favourable pharmacokinetic profile than traditional inorganic forms like SSe, owing to their unique physicochemical properties and passive uptake mechanisms (Li et al. 2024, Loeschner et al. 2014). Our results corroborate these findings, confirming that the nutritional strategy was successful in elevating selenium status in both dams and their offspring during the critical transition period.

The central finding of this study, the significant downregulation of HSP transcription in selenium‐supplemented groups, is consistent with successful mitigation of cellular stress, although direct measurements of antioxidant enzymes would be needed to confirm this mechanism. The periparturient period is characterised by heightened metabolic activity and oxidative stress, which typically induces a strong upregulation of HSPs as a protective mechanism (Berestoviy et al. 2021, Yaghoobpour et al. 2025). The fact that this upregulation was markedly blunted in the SeNP and SSe groups, with SeNPs showing a more potent effect, raises the possibility that selenium supplementation bolstered the endogenous antioxidant defence system, an interpretation supported by the negative correlation between serum selenium and HSP transcription. (Barchielli et al. 2022). This is consistent with the well‐established function of selenoenzymes, such as glutathione peroxidase, in neutralising reactive oxygen species (ROS), thereby pre‐empting the protein damage that would otherwise trigger a stress response and HSP induction (Ikwegbue et al. 2017). The mechanistic basis for this attenuation likely involves the heat shock factor 1 (*HSF1*) pathway. Under normal conditions, *HSF1* is maintained in an inactive state through binding with *HSP90*. Oxidative stress generates misfolded proteins, which titrate *HSP90* away from *HSF1*, allowing *HSF1* trimerisation, nuclear translocation and subsequent transcription of *HSP* genes. By reducing ROS burden through enhanced selenoenzyme activity, selenium supplementation may lower the concentration of misfolded proteins, thereby diminishing the need for *HSF1* activation and *HSP* transcription (Prodromou 2016). Our results are in agreement with studies in other species, where selenium supplementation has been shown to attenuate HSP expression under various stress conditions, linking improved antioxidant capacity to a reduced cellular demand for molecular chaperones (Aderao et al. 2023, Kumbhar et al. 2018).

However, some studies have reported a paradoxical upregulation of HSPs following selenium supplementation, particularly under conditions of selenium deficiency or in specific tissues. This conflict may be explained by the ‘dual‐role’ of selenium and the concept of hormesis (Muszyńska and Labudda 2019). At optimal levels, selenium enhances antioxidant capacity, mitigating stress and the need for HSP induction (Malyar et al. 2020). In contrast, in deficiency, or at supranutritional/toxic doses, selenium itself can act as a pro‐oxidant or fail to control redox balance, thereby inducing oxidative stress and subsequently upregulating HSPs (Kondaparthi et al. 2019). The significant negative correlation we observed between serum selenium levels and HSP transcription strongly supports the former scenario, positioning our findings within the successful paradigm where selenium adequacy quenches the triggers of the heat shock response. This inverse relationship is mechanistically plausible: higher selenium availability increases synthesis of GPx and other selenoproteins, which metabolise hydrogen peroxide and lipid hydroperoxides (Zoidis et al. 2018). Lower ROS levels reduce oxidative damage to nascent polypeptides, preserving proteostasis and minimising the accumulation of misfolded proteins that would otherwise trigger HSF1‐mediated HSP upregulation (Maty 2023). Our findings are consistent with recent mechanistic studies in pregnant rats. Manojlović‐Stojanoski et al. (2022) demonstrated that equivalent doses of SeNPs and sodium selenite produce redox‐state dependent effects on placental oxidative stress parameters, with SeNPs showing higher tissue accumulation but differential effects on antioxidant enzymes. Similarly, Manojlović‐Stojanoski et al. (2024) reported that the two selenium forms differentially modulate maternal and foetal liver antioxidant responses in a sex‐specific manner, with SeNPs exhibiting lower pro‐oxidant activity than selenite. These mechanistic insights support our interpretation that SeNP supplementation enhances antioxidant capacity, thereby reducing the cellular stress burden reflected by suppressed HSP transcription.

A notable finding was the absence of significant differences in either serum selenium or HSP transcript levels between cloned and non‐cloned animals. This conflicts with the well‐documented literature on the physiological vulnerabilities of SCNT‐derived animals, often attributed to epigenetic aberrations and altered stress responses. One potential explanation for this lack of difference is the specific focus on leukocytes in the peripheral blood. It is possible that the systemic stress response measured in circulating cells does not fully capture tissue‐specific dysregulations that may be present in the placenta or key foetal organs of cloned conceptuses (Gunawardana et al. 2021). Alternatively, the rigorous health screening and uniform management conditions in this study may have minimised phenotypic disparities, or the chosen HSP genes may not be the primary markers of the unique stresses associated with cloning. This finding suggests that under optimised nutritional and management regimes, the leukocyte stress response in periparturient cloned goats can be comparable to that of their non‐cloned counterparts (Wells 2006).

## 5 Conclusion

This study raises the possibility that selenium supplementation, especially in its nanoform, can enhance the antioxidant status of periparturient dams and passively transfer this benefit to their offspring, as evidenced by elevated serum selenium and a concomitant reduction in the transcription of key HSP genes. This indicates a mitigation of the systemic oxidative stress inherent to the transition period. The superior efficacy of SeNPs over SSe underscores the importance of selenium source in nutritional strategies. While the lack of a clear cloning effect is present, it highlights the potential for nutritional interventions to ameliorate physiological stressors, potentially masking inherent vulnerabilities. Future research should focus on tissue‐specific HSP expression and a broader panel of oxidative stress markers to further elucidate the molecular mechanisms by which nano‐selenium supports maternal and neonatal health in both conventional and biotechnologically derived animals.

## Author Contributions

**Gholam‐Ali Kojouri**: writing – original draft, software, methodology, investigation, formal analysis, supervision, resources, project administration, data curation. **Hossein Hassanpour**: methodology, writing – review and editing. **Ahmad Yousefi**: validation, data curation, methodology, investigation, conceptualization.

## Funding

The authors have nothing to report.

## Ethics Statement

The study underwent an ethical review and was approved (approved code: IR.IAU.K.REC.1400.1101) by the Institutional Animal Care and Use Committee of Shahrekord University under the standard of the 1964 Declaration of Helsinki, and was carried out at the experimental facility of this university.

## Conflicts of Interest

The authors declare no conflicts of interest.

## Supporting information




**Supporting file 1**: vms371135‐sup‐0001‐TableS1.docx

## Data Availability

Data will be made available on request.
