# Supplementary material for: Transcriptional Regulation of HSP60, HSP70, and HSP90 in Response to Selenium Nanoparticles and Sodium Selenite in Periparturient Cloned Saanen Goats and Their Offspring
Source: Vet Med Sci. 2026 Jul 29;12(5):e71135. doi: 10.1002/vms3.71135 (PMC13418857; doi:10.1002/vms3.71135)
Supplement: Supplementary file 1 — Supporting file 1: vms371135‐sup‐0001‐TableS1.docx [file VMS3-12-e71135-s001.docx]

| **Periods** | **Cloned/Non-cloned** | **HSP60** | **HSP70** | **HSP90** |
| --- | --- | --- | --- | --- |
| **Dams** | | | | |
| **Day -14** | **Cloned** | 1.67 ± 0.005 | 0.22 ± 0.002 | 2.21 ± 0.005 |
|  | **Non-cloned** | 1.65 ± 0.007 | 0.23 ± 0.004 | 2.21 ± 0.010 |
| **Day -7** | **Cloned** | 1.67 ± 0.003 | 0.23 ± 0.006 | 2.21 ± 0.007 |
|  | **Non-cloned** | 1.66 ± 0.004 | 0.23 ± 0.005 | 2.22 ± 0.007 |
| **Day 0 (Parturition)** | **Cloned** | 1.66 ± 0.004 | 0.29 ± 0.022 | 3.24 ± 0.333 |
|  | **Non-cloned** | 1.67 ± 0.005 | 0.29 ± 0.026 | 2.99 ± 0.317 |
| **Day +7** | **Cloned** | 1.77 ± 0.020 | 0.23 ± 0.006 | 2.30 ± 0.015 |
|  | **Non-cloned** | 1.78 ± 0.021 | 0.23 ± 0.003 | 2.29 ± 0.021 |
| **Day +14** | **Cloned** | 1.78 ± 0.022 | 0.22 ± 0.005 | 2.22 ± 0.008 |
|  | **Non-cloned** | 1.78 ± 0.018 | 0.23 ± 0.004 | 2.21 ± 0.006 |
| **Neonates of cloned and non-cloned dams** | | | | |
| **Day 0 (Birth)** | **Cloned** | 1.50 ± 0.010 | 0.29 ± 0.012 | 3.02 ± 0.255 |
|  | **Non-cloned** | 1.51 ± 0.007 | 0.28 ± 0.009 | 2.88 ± 0.197 |
| **Day +7** | **Cloned** | 1.74 ± 0.030 | 0.25 ± 0.006 | 2.15 ± 0.005 |
|  | **Non-cloned** | 1.72 ± 0.023 | 0.25 ± 0.005 | 2.15 ± 0.006 |
| **Day +14** | **Cloned** | 1.74 ± 0.030 | 0.19 ± 0.002 | 2.12 ± 0.004 |
|  | **Non-cloned** | 1.73 ± 0.023 | 0.19 ± 0.002 | 2.12 ± 0.300 |

**Table S1**. HSPs gene expression (/GAPDH) in leukocytes of cloned and non-cloned goats and their neonates at different time points.

Data are as mean ± standard error.
